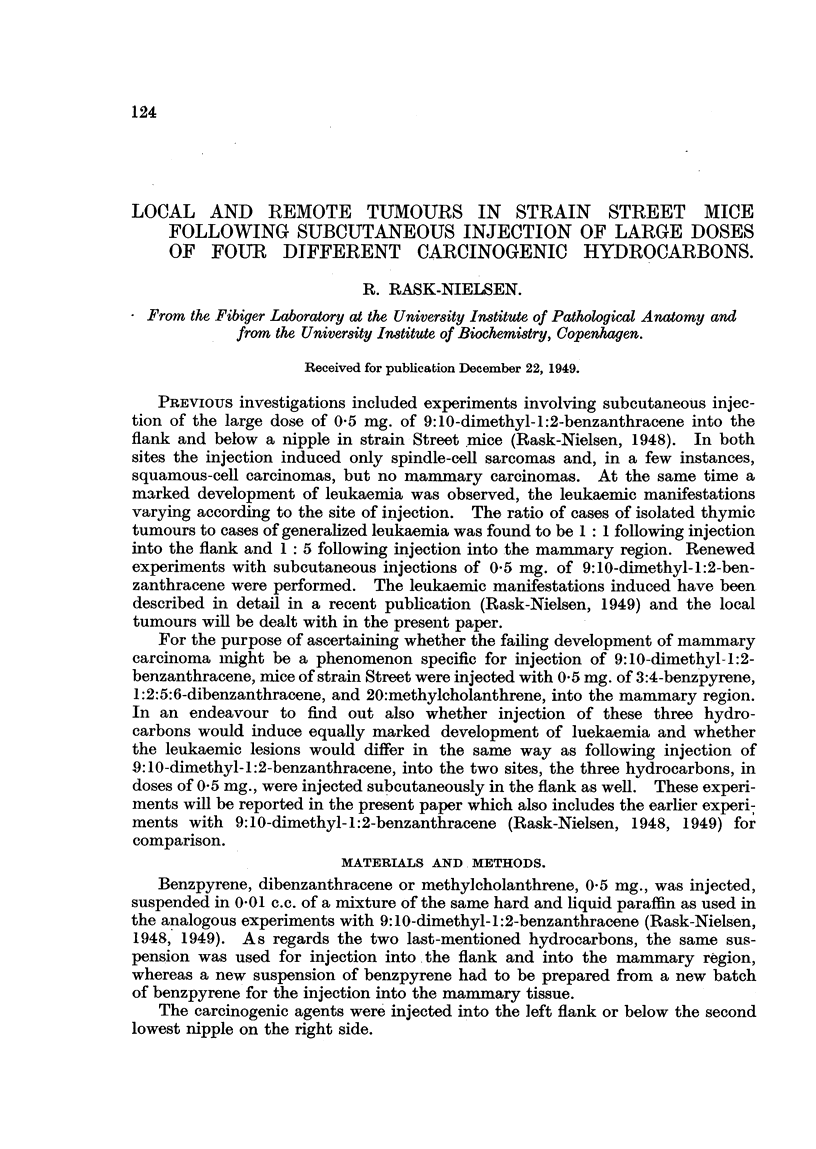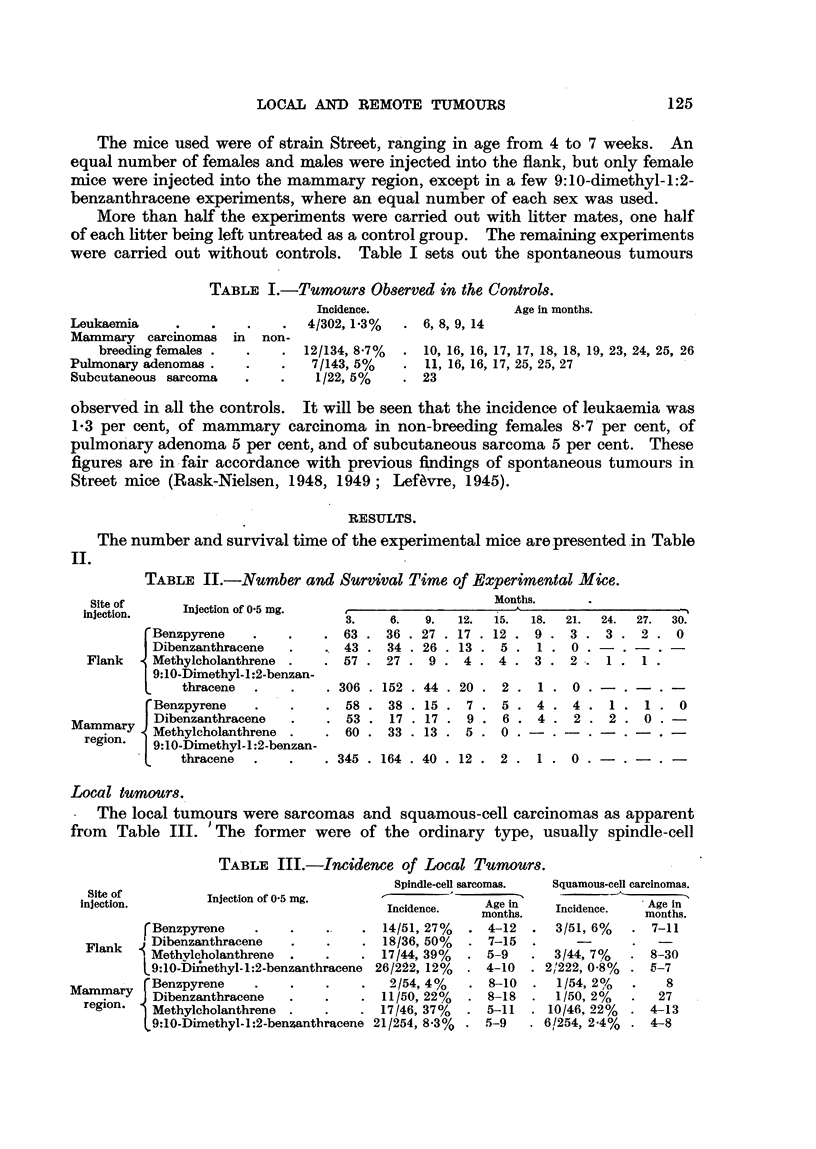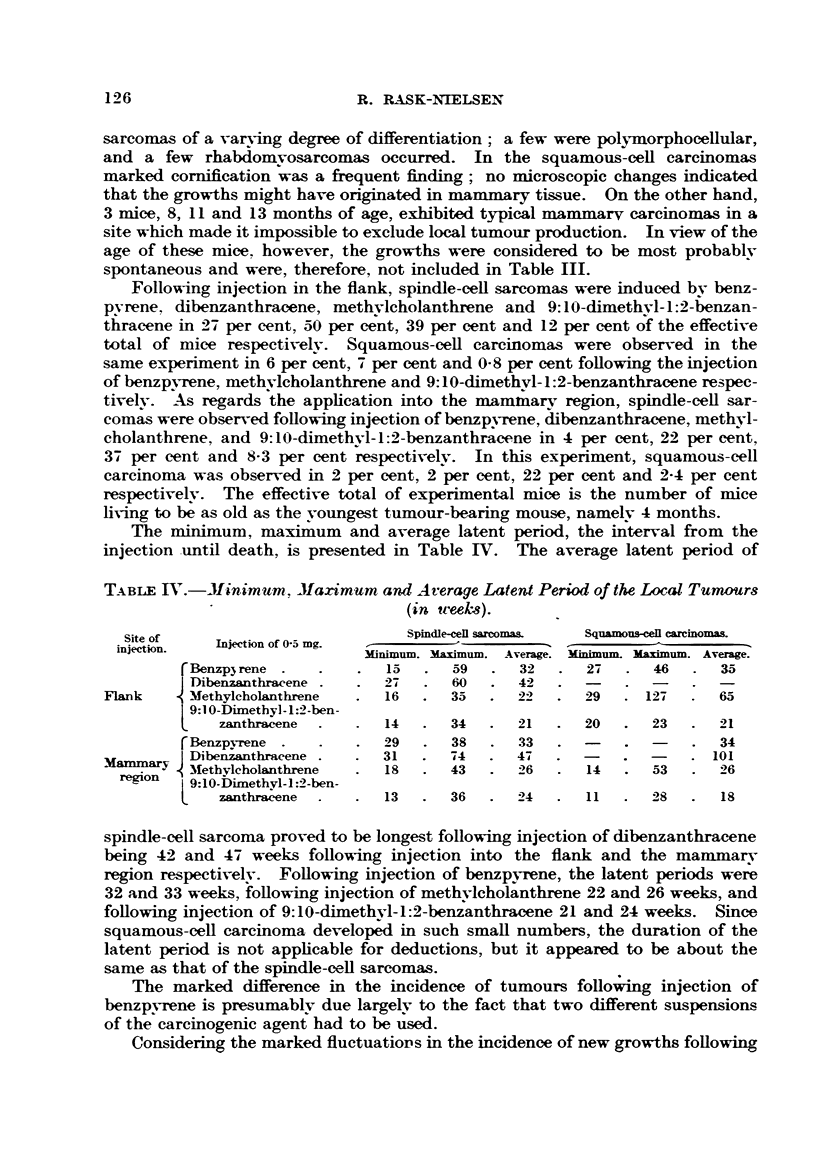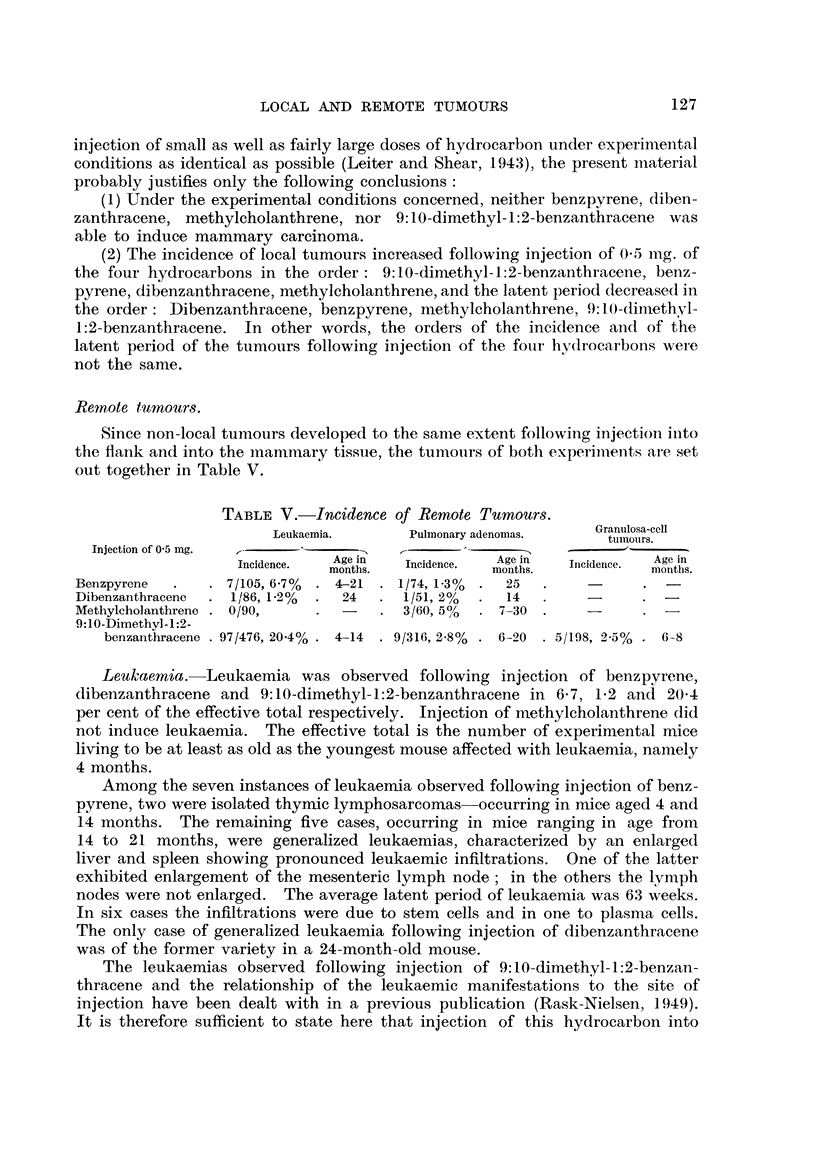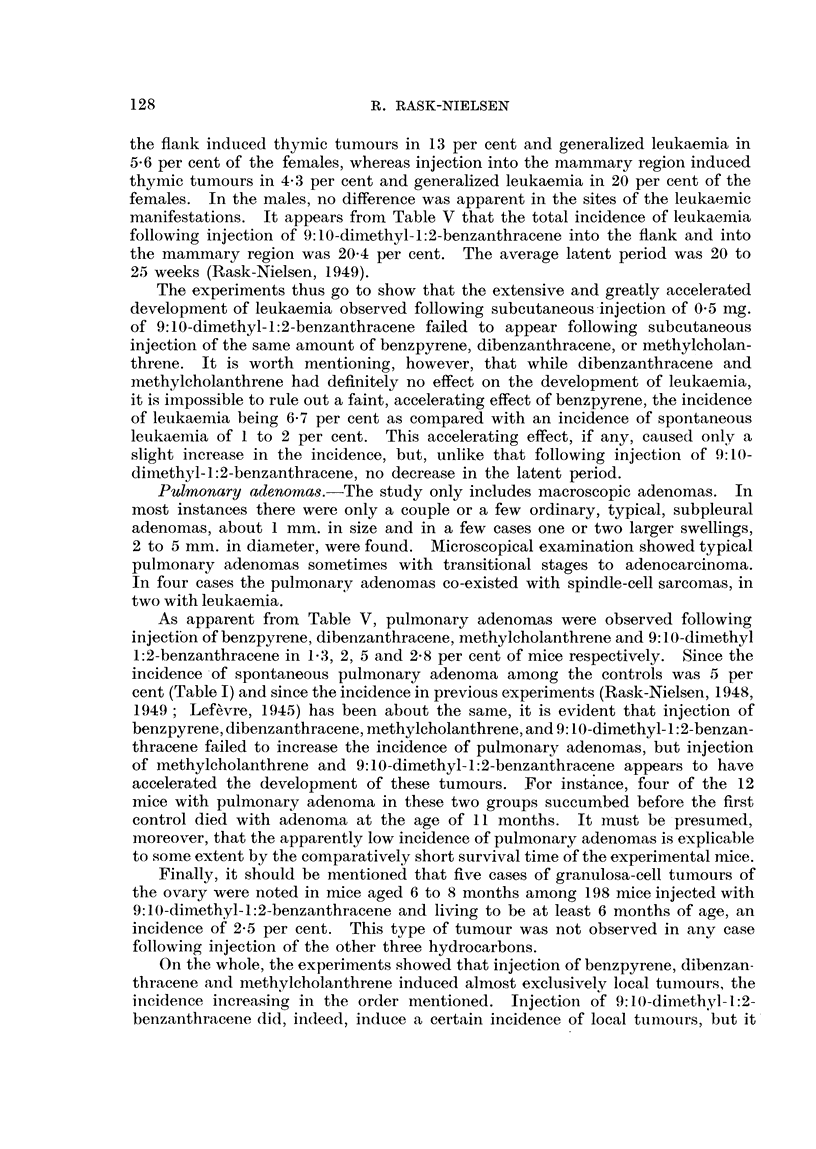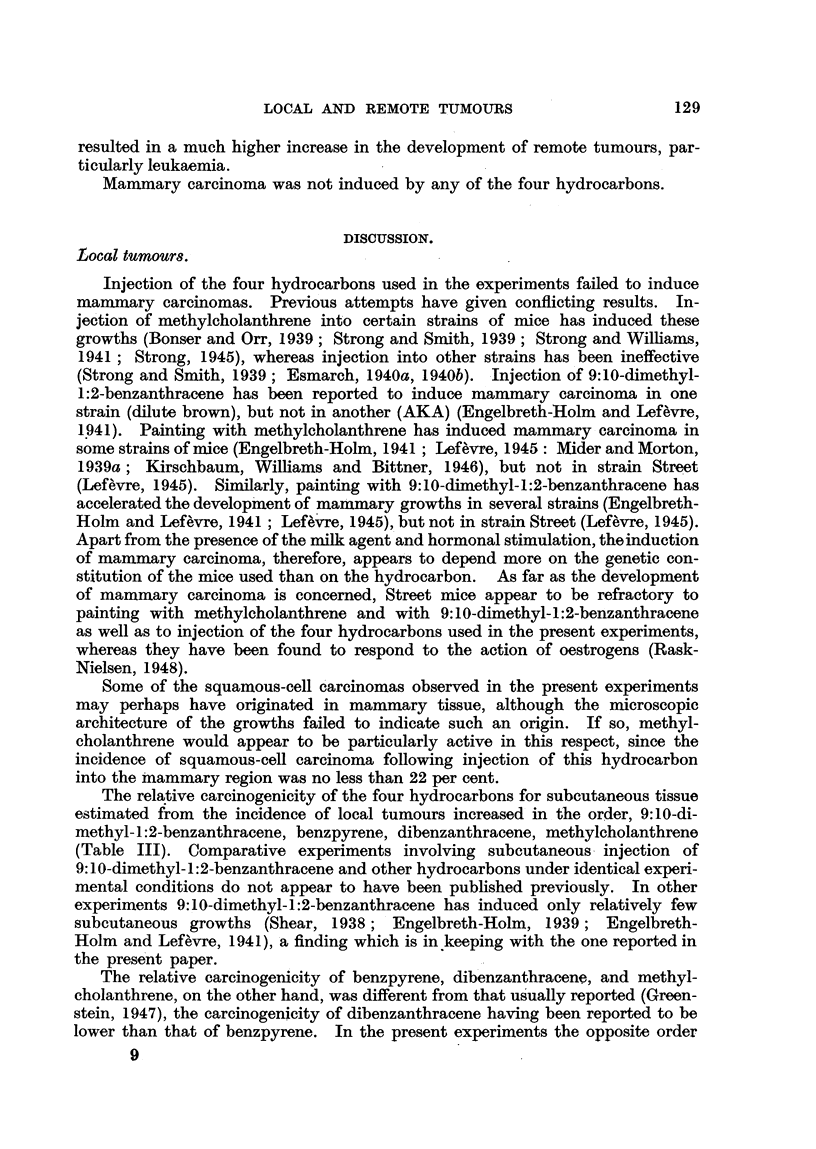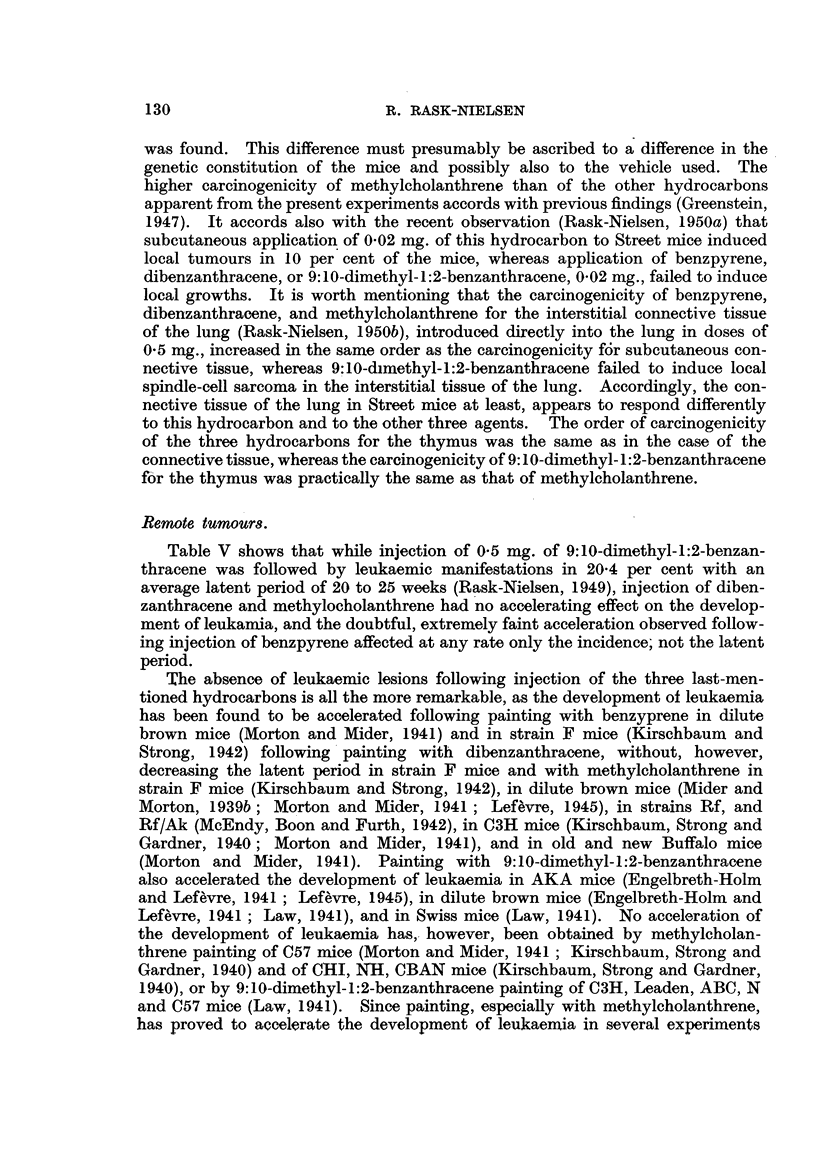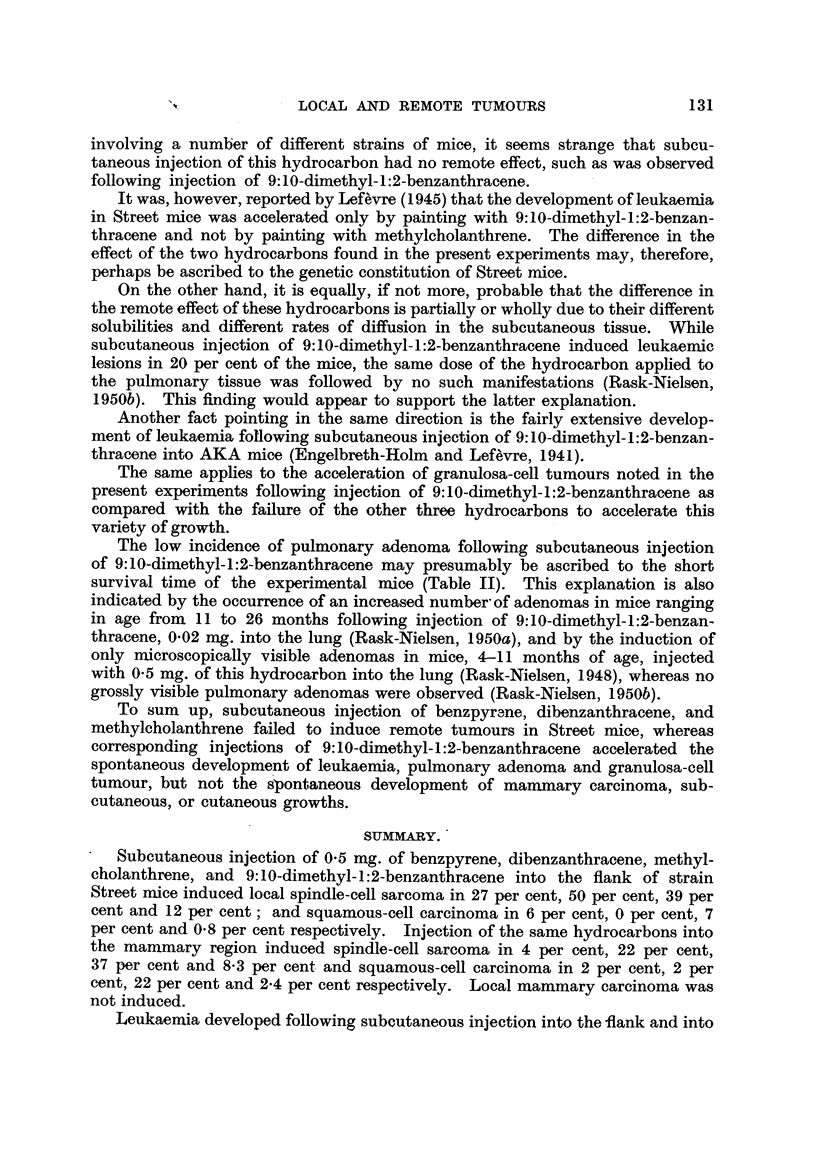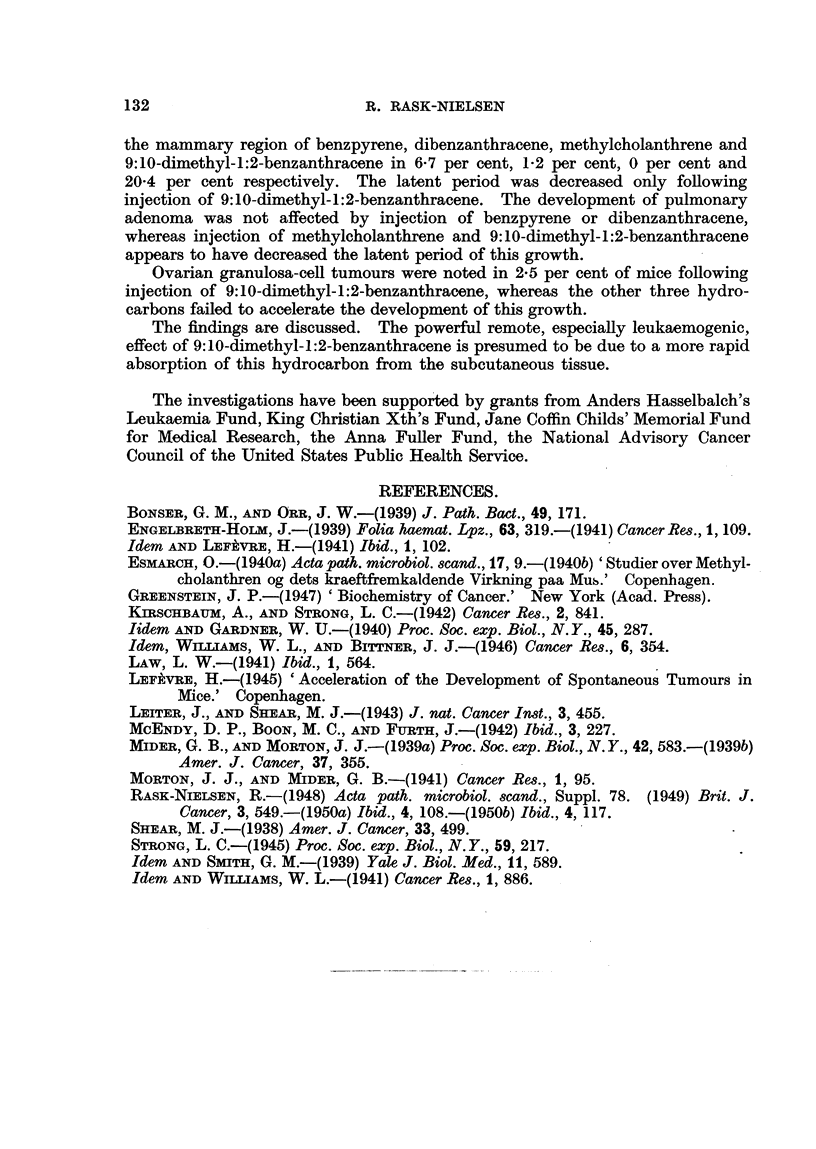# Local and Remote Tumours in Strain Street Mice following Subcutaneous Injection of Large Doses of Four Different Carcinogenic Hydrocarbons

**DOI:** 10.1038/bjc.1950.12

**Published:** 1950-03

**Authors:** R. Rask-Nielsen


### LOCAL AND REMOTE TUMOURS IN STRAIN STREET MICE FOLLOWING SUBCUTANEOUS INJECTION OF LARGE DOSES OF FOUR DIFFERENT CARCINOGENIC HYDROCARBONS.

R. RASK-NIELSEN.

*From the Fibiger Laboratory at the University Institute of Pathological Anatomy and from the University Institute of Biochemistry, Copenhagen.*

Received for publication December 22, 1949.

PREVIOUS investigations included experiments involving subcutaneous injection of the large dose of 0·5 mg. of 9:10-dimethyl-1:2-benzanthracene into the flank and below a nipple in strain Street mice (Rask-Nielsen, 1948). In both sites the injection induced only spindle-cell sarcomas and, in a few instances, squamous-cell carcinomas, but no mammary carcinomas. At the same time a marked development of leukaemia was observed, the leukaemic manifestations varying according to the site of injection. The ratio of cases of isolated thymic tumours to cases of generalized leukaemia was found to be 1 : 1 following injection into the flank and 1 : 5 following injection into the mammary region. Renewed experiments with subcutaneous injections of 0·5 mg. of 9:10-dimethyl-1:2-benzanthracene were performed. The leukaemic manifestations induced have been described in detail in a recent publication (Rask-Nielsen, 1949) and the local tumours will be dealt with in the present paper.

For the purpose of ascertaining whether the failing development of mammary carcinoma might be a phenomenon specific for injection of 9:10-dimethyl-1:2-benzanthracene, mice of strain Street were injected with 0·5 mg. of 3:4-benzpyrene, 1:2:5:6-dibenzanthracene, and 20:methylcholanthrene, into the mammary region. In an endeavour to find out also whether injection of these three hydrocarbons would induce equally marked development of luekaemia and whether the leukaemic lesions would differ in the same way as following injection of 9:10-dimethyl-1:2-benzanthracene, into the two sites, the three hydrocarbons, in doses of 0·5 mg., were injected subcutaneously in the flank as well. These experiments will be reported in the present paper which also includes the earlier experiments with 9:10-dimethyl-1:2-benzanthracene (Rask-Nielsen, 1948, 1949) for comparison.

### MATERIALS AND METHODS.

Benzpyrene, dibenzanthracene or methylcholanthrene, 0·5 mg., was injected, suspended in 0·01 c.c. of a mixture of the same hard and liquid paraffin as used in the analogous experiments with 9:10-dimethyl-1:2-benzanthracene (Rask-Nielsen, 1948, 1949). As regards the two last-mentioned hydrocarbons, the same suspension was used for injection into the flank and into the mammary region, whereas a new suspension of benzpyrene had to be prepared from a new batch of benzpyrene for the injection into the mammary tissue.

The carcinogenic agents were injected into the left flank or below the second lowest nipple on the right side.

The mice used were of strain Street, ranging in age from 4 to 7 weeks. An equal number of females and males were injected into the flank, but only female mice were injected into the mammary region, except in a few 9:10-dimethyl-1:2-benzanthracene experiments, where an equal number of each sex was used.

More than half the experiments were carried out with litter mates, one half of each litter being left untreated as a control group. The remaining experiments were carried out without controls. Table I sets out the spontaneous tumours observed in all the controls. It will be seen that the incidence of leukaemia was 1·3 per cent, of mammary carcinoma in non-breeding females 8·7 per cent, of pulmonary adenoma 5 per cent, and of subcutaneous sarcoma 5 per cent. These figures are in fair accordance with previous findings of spontaneous tumours in Street mice (Rask-Nielsen, 1948, 1949; Lefèvre, 1945).

TABLE I.—*Tumours Observed in the Controls.*

| | Incidence. | Age in months. |
|---|---|---|
| Leukaemia | 4/302, 1·3% | 6, 8, 9, 14 |
| Mammary carcinomas in non-breeding females | 12/134, 8·7% | 10, 16, 16, 17, 17, 18, 18, 19, 23, 24, 25, 26 |
| Pulmonary adenomas | 7/143, 5% | 11, 16, 16, 17, 25, 25, 27 |
| Subcutaneous sarcoma | 1/22, 5% | 23 |

RESULTS.

The number and survival time of the experimental mice are presented in Table II.

TABLE II.—*Number and Survival Time of Experimental Mice.*

| Site of injection. | Injection of 0·5 mg. | Months. 3. | 6. | 9. | 12. | 15. | 18. | 21. | 24. | 27. | 30. |
|---|---|---|---|---|---|---|---|---|---|---|---|
| Flank | Benzpyrene | 63 | 36 | 27 | 17 | 12 | 9 | 3 | 3 | 2 | 0 |
| | Dibenzanthracene | 43 | 34 | 26 | 13 | 5 | 1 | 0 | — | — | — |
| | Methylcholanthrene | 57 | 27 | 9 | 4 | 4 | 3 | 2 | 1 | 1 | |
| | 9:10-Dimethyl-1:2-benzanthracene | 306 | 152 | 44 | 20 | 2 | 1 | 0 | — | — | — |
| Mammary region. | Benzpyrene | 58 | 38 | 15 | 7 | 5 | 4 | 4 | 1 | 1 | 0 |
| | Dibenzanthracene | 53 | 17 | 17 | 9 | 6 | 4 | 2 | 2 | 0 | — |
| | Methylcholanthrene | 60 | 33 | 13 | 5 | 0 | — | — | — | — | — |
| | 9:10-Dimethyl-1:2-benzanthracene | 345 | 164 | 40 | 12 | 2 | 1 | 0 | — | — | — |

*Local tumours.*

The local tumours were sarcomas and squamous-cell carcinomas as apparent from Table III. The former were of the ordinary type, usually spindle-cell

TABLE III.—*Incidence of Local Tumours.*

| Site of injection. | Injection of 0·5 mg. | Spindle-cell sarcomas. Incidence. | Age in months. | Squamous-cell carcinomas. Incidence. | Age in months. |
|---|---|---|---|---|---|
| Flank | Benzpyrene | 14/51, 27% | 4–12 | 3/51, 6% | 7–11 |
| | Dibenzanthracene | 18/36, 50% | 7–15 | — | — |
| | Methylcholanthrene | 17/44, 39% | 5–9 | 3/44, 7% | 8–30 |
| | 9:10-Dimethyl-1:2-benzanthracene | 26/222, 12% | 4–10 | 2/222, 0·8% | 5–7 |
| Mammary region. | Benzpyrene | 2/54, 4% | 8–10 | 1/54, 2% | 8 |
| | Dibenzanthracene | 11/50, 22% | 8–18 | 1/50, 2% | 27 |
| | Methylcholanthrene | 17/46, 37% | 5–11 | 10/46, 22% | 4–13 |
| | 9:10-Dimethyl-1:2-benzanthracene | 21/254, 8·3% | 5–9 | 6/254, 2·4% | 4–8 |

sarcomas of a varying degree of differentiation ; a few were polymorphocellular, and a few rhabdomyosarcomas occurred. In the squamous-cell carcinomas marked cornification was a frequent finding ; no microscopic changes indicated that the growths might have originated in mammary tissue. On the other hand, 3 mice, 8, 11 and 13 months of age, exhibited typical mammary carcinomas in a site which made it impossible to exclude local tumour production. In view of the age of these mice, however, the growths were considered to be most probably spontaneous and were, therefore, not included in Table III.

Following injection in the flank, spindle-cell sarcomas were induced by benzpyrene, dibenzanthracene, methylcholanthrene and 9:10-dimethyl-1:2-benzanthracene in 27 per cent, 50 per cent, 39 per cent and 12 per cent of the effective total of mice respectively. Squamous-cell carcinomas were observed in the same experiment in 6 per cent, 7 per cent and 0·8 per cent following the injection of benzpyrene, methylcholanthrene and 9:10-dimethyl-1:2-benzanthracene respectively. As regards the application into the mammary region, spindle-cell sarcomas were observed following injection of benzpyrene, dibenzanthracene, methylcholanthrene, and 9:10-dimethyl-1:2-benzanthracene in 4 per cent, 22 per cent, 37 per cent and 8·3 per cent respectively. In this experiment, squamous-cell carcinoma was observed in 2 per cent, 2 per cent, 22 per cent and 2·4 per cent respectively. The effective total of experimental mice is the number of mice living to be as old as the youngest tumour-bearing mouse, namely 4 months.

The minimum, maximum and average latent period, the interval from the injection until death, is presented in Table IV. The average latent period of

TABLE IV.—*Minimum, Maximum and Average Latent Period of the Local Tumours (in weeks).*

| Site of injection. | Injection of 0·5 mg. | Spindle-cell sarcomas. | | | Squamous-cell carcinomas. | | |
|---|---|---|---|---|---|---|---|
| | | Minimum. | Maximum. | Average. | Minimum. | Maximum. | Average. |
| Flank | Benzpyrene | 15 | 59 | 32 | 27 | 46 | 35 |
| | Dibenzanthracene | 27 | 60 | 42 | — | — | — |
| | Methylcholanthrene | 16 | 35 | 22 | 29 | 127 | 65 |
| | 9:10-Dimethyl-1:2-benzanthracene | 14 | 34 | 21 | 20 | 23 | 21 |
| Mammary region | Benzpyrene | 29 | 38 | 33 | — | — | 34 |
| | Dibenzanthracene | 31 | 74 | 47 | — | — | 101 |
| | Methylcholanthrene | 18 | 43 | 26 | 14 | 53 | 26 |
| | 9:10-Dimethyl-1:2-benzanthracene | 13 | 36 | 24 | 11 | 28 | 18 |

spindle-cell sarcoma proved to be longest following injection of dibenzanthracene being 42 and 47 weeks following injection into the flank and the mammary region respectively. Following injection of benzpyrene, the latent periods were 32 and 33 weeks, following injection of methylcholanthrene 22 and 26 weeks, and following injection of 9:10-dimethyl-1:2-benzanthracene 21 and 24 weeks. Since squamous-cell carcinoma developed in such small numbers, the duration of the latent period is not applicable for deductions, but it appeared to be about the same as that of the spindle-cell sarcomas.

The marked difference in the incidence of tumours following injection of benzpyrene is presumably due largely to the fact that two different suspensions of the carcinogenic agent had to be used.

Considering the marked fluctuations in the incidence of new growths following injection of small as well as fairly large doses of hydrocarbon under experimental conditions as identical as possible (Leiter and Shear, 1943), the present material probably justifies only the following conclusions :

(1) Under the experimental conditions concerned, neither benzpyrene, dibenzanthracene, methylcholanthrene, nor 9:10-dimethyl-1:2-benzanthracene was able to induce mammary carcinoma.

(2) The incidence of local tumours increased following injection of 0·5 mg. of the four hydrocarbons in the order : 9:10-dimethyl-1:2-benzanthracene, benzpyrene, dibenzanthracene, methylcholanthrene, and the latent period decreased in the order : Dibenzanthracene, benzpyrene, methylcholanthrene, 9:10-dimethyl-1:2-benzanthracene. In other words, the orders of the incidence and of the latent period of the tumours following injection of the four hydrocarbons were not the same.

*Remote tumours.*

Since non-local tumours developed to the same extent following injection into the flank and into the mammary tissue, the tumours of both experiments are set out together in Table V.

TABLE V.—*Incidence of Remote Tumours.*

| Injection of 0·5 mg. | Leukaemia. | | Pulmonary adenomas. | | Granulosa-cell tumours. | |
|---|---|---|---|---|---|---|
| | Incidence. | Age in months. | Incidence. | Age in months. | Incidence. | Age in months. |
| Benzpyrene | 7/105, 6·7% | 4–21 | 1/74, 1·3% | 25 | — | — |
| Dibenzanthracene | 1/86, 1·2% | 24 | 1/51, 2% | 14 | — | — |
| Methylcholanthrene | 0/90, | — | 3/60, 5% | 7–30 | — | — |
| 9:10-Dimethyl-1:2-benzanthracene | 97/476, 20·4% | 4–14 | 9/316, 2·8% | 6–20 | 5/198, 2·5% | 6–8 |

*Leukaemia.*—Leukaemia was observed following injection of benzpyrene, dibenzanthracene and 9:10-dimethyl-1:2-benzanthracene in 6·7, 1·2 and 20·4 per cent of the effective total respectively. Injection of methylcholanthrene did not induce leukaemia. The effective total is the number of experimental mice living to be at least as old as the youngest mouse affected with leukaemia, namely 4 months.

Among the seven instances of leukaemia observed following injection of benzpyrene, two were isolated thymic lymphosarcomas—occurring in mice aged 4 and 14 months. The remaining five cases, occurring in mice ranging in age from 14 to 21 months, were generalized leukaemias, characterized by an enlarged liver and spleen showing pronounced leukaemic infiltrations. One of the latter exhibited enlargement of the mesenteric lymph node ; in the others the lymph nodes were not enlarged. The average latent period of leukaemia was 63 weeks. In six cases the infiltrations were due to stem cells and in one to plasma cells. The only case of generalized leukaemia following injection of dibenzanthracene was of the former variety in a 24-month-old mouse.

The leukaemias observed following injection of 9:10-dimethyl-1:2-benzanthracene and the relationship of the leukaemic manifestations to the site of injection have been dealt with in a previous publication (Rask-Nielsen, 1949). It is therefore sufficient to state here that injection of this hydrocarbon into the flank induced thymic tumours in 13 per cent and generalized leukaemia in 5·6 per cent of the females, whereas injection into the mammary region induced thymic tumours in 4·3 per cent and generalized leukaemia in 20 per cent of the females. In the males, no difference was apparent in the sites of the leukaemic manifestations. It appears from Table V that the total incidence of leukaemia following injection of 9:10-dimethyl-1:2-benzanthracene into the flank and into the mammary region was 20·4 per cent. The average latent period was 20 to 25 weeks (Rask-Nielsen, 1949).

The experiments thus go to show that the extensive and greatly accelerated development of leukaemia observed following subcutaneous injection of 0·5 mg. of 9:10-dimethyl-1:2-benzanthracene failed to appear following subcutaneous injection of the same amount of benzpyrene, dibenzanthracene, or methylcholanthrene. It is worth mentioning, however, that while dibenzanthracene and methylcholanthrene had definitely no effect on the development of leukaemia, it is impossible to rule out a faint, accelerating effect of benzpyrene, the incidence of leukaemia being 6·7 per cent as compared with an incidence of spontaneous leukaemia of 1 to 2 per cent. This accelerating effect, if any, caused only a slight increase in the incidence, but, unlike that following injection of 9:10-dimethyl-1:2-benzanthracene, no decrease in the latent period.

*Pulmonary adenomas.*—The study only includes macroscopic adenomas. In most instances there were only a couple or a few ordinary, typical, subpleural adenomas, about 1 mm. in size and in a few cases one or two larger swellings, 2 to 5 mm. in diameter, were found. Microscopical examination showed typical pulmonary adenomas sometimes with transitional stages to adenocarcinoma. In four cases the pulmonary adenomas co-existed with spindle-cell sarcomas, in two with leukaemia.

As apparent from Table V, pulmonary adenomas were observed following injection of benzpyrene, dibenzanthracene, methylcholanthrene and 9:10-dimethyl 1:2-benzanthracene in 1·3, 2, 5 and 2·8 per cent of mice respectively. Since the incidence of spontaneous pulmonary adenoma among the controls was 5 per cent (Table I) and since the incidence in previous experiments (Rask-Nielsen, 1948, 1949 ; Lefèvre, 1945) has been about the same, it is evident that injection of benzpyrene, dibenzanthracene, methylcholanthrene, and 9:10-dimethyl-1:2-benzanthracene failed to increase the incidence of pulmonary adenomas, but injection of methylcholanthrene and 9:10-dimethyl-1:2-benzanthracene appears to have accelerated the development of these tumours. For instance, four of the 12 mice with pulmonary adenoma in these two groups succumbed before the first control died with adenoma at the age of 11 months. It must be presumed, moreover, that the apparently low incidence of pulmonary adenomas is explicable to some extent by the comparatively short survival time of the experimental mice.

Finally, it should be mentioned that five cases of granulosa-cell tumours of the ovary were noted in mice aged 6 to 8 months among 198 mice injected with 9:10-dimethyl-1:2-benzanthracene and living to be at least 6 months of age, an incidence of 2·5 per cent. This type of tumour was not observed in any case following injection of the other three hydrocarbons.

On the whole, the experiments showed that injection of benzpyrene, dibenzanthracene and methylcholanthrene induced almost exclusively local tumours, the incidence increasing in the order mentioned. Injection of 9:10-dimethyl-1:2-benzanthracene did, indeed, induce a certain incidence of local tumours, but it resulted in a much higher increase in the development of remote tumours, particularly leukaemia.

Mammary carcinoma was not induced by any of the four hydrocarbons.

### DISCUSSION.

*Local tumours.*

Injection of the four hydrocarbons used in the experiments failed to induce mammary carcinomas. Previous attempts have given conflicting results. Injection of methylcholanthrene into certain strains of mice has induced these growths (Bonser and Orr, 1939 ; Strong and Smith, 1939 ; Strong and Williams, 1941 ; Strong, 1945), whereas injection into other strains has been ineffective (Strong and Smith, 1939 ; Esmarch, 1940*a*, 1940*b*). Injection of 9:10-dimethyl-1:2-benzanthracene has been reported to induce mammary carcinoma in one strain (dilute brown), but not in another (AKA) (Engelbreth-Holm and Lefèvre, 1941). Painting with methylcholanthrene has induced mammary carcinoma in some strains of mice (Engelbreth-Holm, 1941 ; Lefèvre, 1945 : Mider and Morton, 1939*a* ; Kirschbaum, Williams and Bittner, 1946), but not in strain Street (Lefèvre, 1945). Similarly, painting with 9:10-dimethyl-1:2-benzanthracene has accelerated the development of mammary growths in several strains (Engelbreth-Holm and Lefèvre, 1941 ; Lefèvre, 1945), but not in strain Street (Lefèvre, 1945). Apart from the presence of the milk agent and hormonal stimulation, the induction of mammary carcinoma, therefore, appears to depend more on the genetic constitution of the mice used than on the hydrocarbon. As far as the development of mammary carcinoma is concerned, Street mice appear to be refractory to painting with methylcholanthrene and with 9:10-dimethyl-1:2-benzanthracene as well as to injection of the four hydrocarbons used in the present experiments, whereas they have been found to respond to the action of oestrogens (Rask-Nielsen, 1948).

Some of the squamous-cell carcinomas observed in the present experiments may perhaps have originated in mammary tissue, although the microscopic architecture of the growths failed to indicate such an origin. If so, methylcholanthrene would appear to be particularly active in this respect, since the incidence of squamous-cell carcinoma following injection of this hydrocarbon into the mammary region was no less than 22 per cent.

The relative carcinogenicity of the four hydrocarbons for subcutaneous tissue estimated from the incidence of local tumours increased in the order, 9:10-dimethyl-1:2-benzanthracene, benzpyrene, dibenzanthracene, methylcholanthrene (Table III). Comparative experiments involving subcutaneous injection of 9:10-dimethyl-1:2-benzanthracene and other hydrocarbons under identical experimental conditions do not appear to have been published previously. In other experiments 9:10-dimethyl-1:2-benzanthracene has induced only relatively few subcutaneous growths (Shear, 1938 ; Engelbreth-Holm, 1939 ; Engelbreth-Holm and Lefèvre, 1941), a finding which is in keeping with the one reported in the present paper.

The relative carcinogenicity of benzpyrene, dibenzanthracene, and methylcholanthrene, on the other hand, was different from that usually reported (Greenstein, 1947), the carcinogenicity of dibenzanthracene having been reported to be lower than that of benzpyrene. In the present experiments the opposite order was found. This difference must presumably be ascribed to a difference in the genetic constitution of the mice and possibly also to the vehicle used. The higher carcinogenicity of methylcholanthrene than of the other hydrocarbons apparent from the present experiments accords with previous findings (Greenstein, 1947). It accords also with the recent observation (Rask-Nielsen, 1950*a*) that subcutaneous application of 0·02 mg. of this hydrocarbon to Street mice induced local tumours in 10 per cent of the mice, whereas application of benzpyrene, dibenzanthracene, or 9:10-dimethyl-1:2-benzanthracene, 0·02 mg., failed to induce local growths. It is worth mentioning that the carcinogenicity of benzpyrene, dibenzanthracene, and methylcholanthrene for the interstitial connective tissue of the lung (Rask-Nielsen, 1950*b*), introduced directly into the lung in doses of 0·5 mg., increased in the same order as the carcinogenicity for subcutaneous connective tissue, whereas 9:10-dimethyl-1:2-benzanthracene failed to induce local spindle-cell sarcoma in the interstitial tissue of the lung. Accordingly, the connective tissue of the lung in Street mice at least, appears to respond differently to this hydrocarbon and to the other three agents. The order of carcinogenicity of the three hydrocarbons for the thymus was the same as in the case of the connective tissue, whereas the carcinogenicity of 9:10-dimethyl-1:2-benzanthracene for the thymus was practically the same as that of methylcholanthrene.

*Remote tumours.*

Table V shows that while injection of 0·5 mg. of 9:10-dimethyl-1:2-benzanthracene was followed by leukaemic manifestations in 20·4 per cent with an average latent period of 20 to 25 weeks (Rask-Nielsen, 1949), injection of dibenzanthracene and methylocholanthrene had no accelerating effect on the development of leukamia, and the doubtful, extremely faint acceleration observed following injection of benzpyrene affected at any rate only the incidence; not the latent period.

The absence of leukaemic lesions following injection of the three last-mentioned hydrocarbons is all the more remarkable, as the development of leukaemia has been found to be accelerated following painting with benzyprene in dilute brown mice (Morton and Mider, 1941) and in strain F mice (Kirschbaum and Strong, 1942) following painting with dibenzanthracene, without, however, decreasing the latent period in strain F mice and with methylcholanthrene in strain F mice (Kirschbaum and Strong, 1942), in dilute brown mice (Mider and Morton, 1939*b* ; Morton and Mider, 1941 ; Lefèvre, 1945), in strains Rf, and Rf/Ak (McEndy, Boon and Furth, 1942), in C3H mice (Kirschbaum, Strong and Gardner, 1940 ; Morton and Mider, 1941), and in old and new Buffalo mice (Morton and Mider, 1941). Painting with 9:10-dimethyl-1:2-benzanthracene also accelerated the development of leukaemia in AKA mice (Engelbreth-Holm and Lefèvre, 1941 ; Lefèvre, 1945), in dilute brown mice (Engelbreth-Holm and Lefèvre, 1941 ; Law, 1941), and in Swiss mice (Law, 1941). No acceleration of the development of leukaemia has, however, been obtained by methylcholanthrene painting of C57 mice (Morton and Mider, 1941 ; Kirschbaum, Strong and Gardner, 1940) and of CHI, NH, CBAN mice (Kirschbaum, Strong and Gardner, 1940), or by 9:10-dimethyl-1:2-benzanthracene painting of C3H, Leaden, ABC, N and C57 mice (Law, 1941). Since painting, especially with methylcholanthrene, has proved to accelerate the development of leukaemia in several experiments involving a number of different strains of mice, it seems strange that subcutaneous injection of this hydrocarbon had no remote effect, such as was observed following injection of 9:10-dimethyl-1:2-benzanthracene.

It was, however, reported by Lefèvre (1945) that the development of leukaemia in Street mice was accelerated only by painting with 9:10-dimethyl-1:2-benzanthracene and not by painting with methylcholanthrene. The difference in the effect of the two hydrocarbons found in the present experiments may, therefore, perhaps be ascribed to the genetic constitution of Street mice.

On the other hand, it is equally, if not more, probable that the difference in the remote effect of these hydrocarbons is partially or wholly due to their different solubilities and different rates of diffusion in the subcutaneous tissue. While subcutaneous injection of 9:10-dimethyl-1:2-benzanthracene induced leukaemic lesions in 20 per cent of the mice, the same dose of the hydrocarbon applied to the pulmonary tissue was followed by no such manifestations (Rask-Nielsen, 1950*b*). This finding would appear to support the latter explanation.

Another fact pointing in the same direction is the fairly extensive development of leukaemia following subcutaneous injection of 9:10-dimethyl-1:2-benzanthracene into AKA mice (Engelbreth-Holm and Lefèvre, 1941).

The same applies to the acceleration of granulosa-cell tumours noted in the present experiments following injection of 9:10-dimethyl-1:2-benzanthracene as compared with the failure of the other three hydrocarbons to accelerate this variety of growth.

The low incidence of pulmonary adenoma following subcutaneous injection of 9:10-dimethyl-1:2-benzanthracene may presumably be ascribed to the short survival time of the experimental mice (Table II). This explanation is also indicated by the occurrence of an increased number of adenomas in mice ranging in age from 11 to 26 months following injection of 9:10-dimethyl-1:2-benzanthracene, 0·02 mg. into the lung (Rask-Nielsen, 1950*a*), and by the induction of only microscopically visible adenomas in mice, 4–11 months of age, injected with 0·5 mg. of this hydrocarbon into the lung (Rask-Nielsen, 1948), whereas no grossly visible pulmonary adenomas were observed (Rask-Nielsen, 1950*b*).

To sum up, subcutaneous injection of benzpyrene, dibenzanthracene, and methylcholanthrene failed to induce remote tumours in Street mice, whereas corresponding injections of 9:10-dimethyl-1:2-benzanthracene accelerated the spontaneous development of leukaemia, pulmonary adenoma and granulosa-cell tumour, but not the spontaneous development of mammary carcinoma, subcutaneous, or cutaneous growths.

SUMMARY.

Subcutaneous injection of 0·5 mg. of benzpyrene, dibenzanthracene, methylcholanthrene, and 9:10-dimethyl-1:2-benzanthracene into the flank of strain Street mice induced local spindle-cell sarcoma in 27 per cent, 50 per cent, 39 per cent and 12 per cent ; and squamous-cell carcinoma in 6 per cent, 0 per cent, 7 per cent and 0·8 per cent respectively. Injection of the same hydrocarbons into the mammary region induced spindle-cell sarcoma in 4 per cent, 22 per cent, 37 per cent and 8·3 per cent and squamous-cell carcinoma in 2 per cent, 2 per cent, 22 per cent and 2·4 per cent respectively. Local mammary carcinoma was not induced.

Leukaemia developed following subcutaneous injection into the flank and into the mammary region of benzpyrene, dibenzanthracene, methylcholanthrene and 9:10-dimethyl-1:2-benzanthracene in 6·7 per cent, 1·2 per cent, 0 per cent and 20·4 per cent respectively. The latent period was decreased only following injection of 9:10-dimethyl-1:2-benzanthracene. The development of pulmonary adenoma was not affected by injection of benzpyrene or dibenzanthracene, whereas injection of methylcholanthrene and 9:10-dimethyl-1:2-benzanthracene appears to have decreased the latent period of this growth.

Ovarian granulosa-cell tumours were noted in 2·5 per cent of mice following injection of 9:10-dimethyl-1:2-benzanthracene, whereas the other three hydrocarbons failed to accelerate the development of this growth.

The findings are discussed. The powerful remote, especially leukaemogenic, effect of 9:10-dimethyl-1:2-benzanthracene is presumed to be due to a more rapid absorption of this hydrocarbon from the subcutaneous tissue.

The investigations have been supported by grants from Anders Hasselbalch's Leukaemia Fund, King Christian Xth's Fund, Jane Coffin Childs' Memorial Fund for Medical Research, the Anna Fuller Fund, the National Advisory Cancer Council of the United States Public Health Service.

### REFERENCES.

Bonser, G. M., and Orr, J. W.—(1939) *J. Path. Bact.*, **49**, 171.
Engelbreth-Holm, J.—(1939) *Folia haemat. Lpz.*, **63**, 319.—(1941) *Cancer Res.*, **1**, 109.
*Idem* and Lefèvre, H.—(1941) *Ibid.*, **1**, 102.
Esmarch, O.—(1940*a*) *Acta path. microbiol. scand.*, **17**, 9.—(1940*b*) 'Studier over Methylcholanthren og dets kraeftfremkaldende Virkning paa Mus.' Copenhagen.
Greenstein, J. P.—(1947) 'Biochemistry of Cancer.' New York (Acad. Press).
Kirschbaum, A., and Strong, L. C.—(1942) *Cancer Res.*, **2**, 841.
*Iidem* and Gardner, W. U.—(1940) *Proc. Soc. exp. Biol., N.Y.*, **45**, 287.
*Idem*, Williams, W. L., and Bittner, J. J.—(1946) *Cancer Res.*, **6**, 354.
Law, L. W.—(1941) *Ibid.*, **1**, 564.
Lefèvre, H.—(1945) 'Acceleration of the Development of Spontaneous Tumours in Mice.' Copenhagen.
Leiter, J., and Shear, M. J.—(1943) *J. nat. Cancer Inst.*, **3**, 455.
McEndy, D. P., Boon, M. C., and Furth, J.—(1942) *Ibid.*, **3**, 227.
Mider, G. B., and Morton, J. J.—(1939*a*) *Proc. Soc. exp. Biol., N.Y.*, **42**, 583.—(1939*b*) *Amer. J. Cancer*, **37**, 355.
Morton, J. J., and Mider, G. B.—(1941) *Cancer Res.*, **1**, 95.
Rask-Nielsen, R.—(1948) *Acta path. microbiol. scand.*, Suppl. 78. (1949) *Brit. J. Cancer*, **3**, 549.—(1950*a*) *Ibid.*, **4**, 108.—(1950*b*) *Ibid.*, **4**, 117.
Shear, M. J.—(1938) *Amer. J. Cancer*, **33**, 499.
Strong, L. C.—(1945) *Proc. Soc. exp. Biol., N.Y.*, **59**, 217.
*Idem* and Smith, G. M.—(1939) *Yale J. Biol. Med.*, **11**, 589.
*Idem* and Williams, W. L.—(1941) *Cancer Res.*, **1**, 886.